# Compensation Techniques for Photosensors Used in High-Precision Accelerometers

**DOI:** 10.3390/mi15091131

**Published:** 2024-09-05

**Authors:** Yuan Wei, Jianhua Yang, Pengfei Li, Junling Zhang, Pu Liang

**Affiliations:** 1 School of Automation, Northwestern Polytechnical University, Xi’an 710129, China; buaaweiyuan@126.com (Y.W.); leguangren@163.com (J.Z.); 2 School of Marine Science and Technology, Northwestern Polytechnical University, Xi’an 710129, China; pfcrazy@163.com; 3 AVIC Xi’an Flight Automatic Control Research Institute, Xi’an 710065, China; lp_dut@163.com

**Keywords:** photosensors, compensation, accelerometer

## Abstract

Temperature exerts a profound influence on the fidelity of photosensors, making the attainment of reliable temperature compensation a formidable task within engineering realms. This research delves into the intricacies of photosensors used in high-precision accelerometers, proposing an innovative, high-precision, adaptive, closed-loop compensation mechanism. Our design stands in stark contrast to traditional open-loop models, demonstrating superior performance by achieving a remarkable reduction in compensation error—nearly 98%. This advancement in consistency and precision marks a significant leap forward for the application of high-precision photosensors in engineering contexts.

## 1. Introduction

Photosensors, celebrated for their non-destructive and high-precision capabilities in detecting a spectrum of physical quantities, owe their widespread adoption to attributes such as non-contact operation, heightened sensitivity, and swift response times [1,2,3,4]. High-precision accelerometers, epitomizing motion-based sensors, boast displacement detection accuracies reaching into the micrometer and sub-micrometer scales. The pivot to photosensors from traditional capacitive and inductive sensors marks a cutting-edge trend in the quest for enhanced detection precision [5,6,7]. Despite these advancements, the susceptibility of photosensors’ optical traits to temperature variations poses a significant challenge, often introducing measurement discrepancies that impede accuracy and reliability in engineering contexts. Hence, the imperative to govern light intensity stability across a broad temperature spectrum is paramount for the realization of high-precision measurements with photosensitive sensors.

The quest to enhance the intrinsic performance of photosensors has spurred a multitude of innovative approaches. One such technique involves the utilization of wavelength-dependent filters to alter the architecture of light-emitting diodes (LEDs). This strategic modification aims to mitigate the LED’s temperature coefficient, thereby curtailing the temperature-induced perturbations on light output intensity. Nonetheless, this comes at the cost of a potential decline in the LED’s overall luminous efficacy [8]. Additionally, the susceptibility of photosensors to temperature fluctuations can be mitigated through refined packaging and welding methods. These techniques ensure that temperature variations are kept within a more confined threshold [9,10]. While effective in reducing temperature-induced changes, these methods are limited in scope, being effective only within a specific range, and cannot entirely negate the impact of temperature.

Alternative strategies concentrate on regulating the ambient temperature surrounding the sensors to foster thermal equilibrium. For instance, one approach involves mounting the photosensor on a substrate with substantial heat capacity, which serves to dampen the intensity oscillations triggered by temperature shifts. However, this method is noted for its relatively modest precision [11]. A more sophisticated method necessitates an enclosed temperature-control apparatus to maintain a stable thermal environment for the photosensor [12,13,14,15,16]. This approach, while effective, is contingent upon a multi-tiered temperature regulation system, superior thermal insulation, and accurate temperature gauging, culminating in a more elaborate and bulkier system. The prevalent method of counteracting temperature effects is through compensatory measures that oppose the direction of impact. Leveraging the thermal properties of certain materials, the composition or surface treatment of the photosensor can be engineered to mitigate or neutralize the material property alterations that accompany temperature changes [17,18]. The deployment of temperature sensors to monitor the real-time ambient temperature surrounding the light source allows for the dynamic modulation of the driving current through the control circuit. This, in turn, facilitates temperature compensation, with ongoing research dedicated to refining the precision of this process [19,20,21,22,23].

In essence, while various methods have been explored to refine the performance of photosensors, each comes with its own set of trade-offs and applications. The ongoing evolution of these techniques is crucial for the advancement of photosensor technology, particularly in high-precision applications where temperature stability is paramount. In this study, various compensation schemes for photosensors used in high-precision accelerometer were explored. A closed-loop compensation scheme based on light intensity detection was proposed for the first time and comprehensively compared with open-loop first- and second-order compensation schemes. The proposed model was verified by conducting simulations and experiments.

## 2. Photosensors

**Figure 1:**
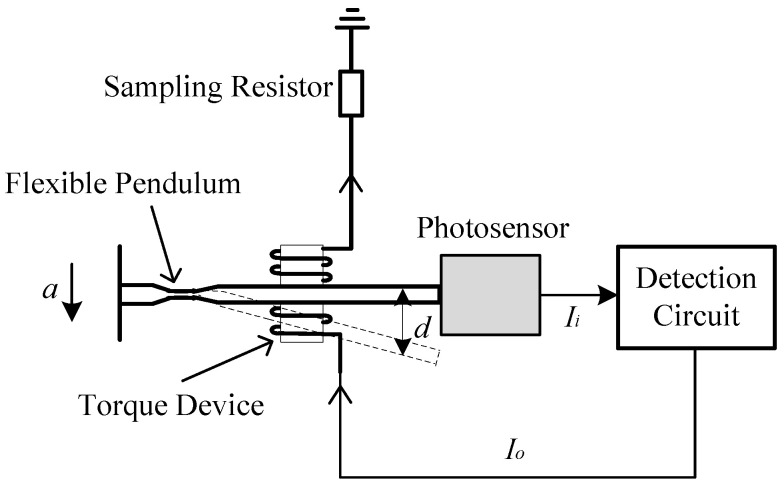
Composition of high-precision optical accelerometer.

The photosensor studied in this paper is an important part of the high-precision optical accelerometer. Figure 1 shows the composition of the high-precision optical accelerometer, which contains a flexible pendulum, a photosensor, a torque device and a detection circuit. When the acceleration *a* is generated, the flexible pendulum moves upward or downward under the action of *a*, generating the displacement *d*. The photosensor can transmit and receive optical signals, convert the displacement information into an optical signal *P*, and further convert it into an electrical signal *I_i_*. The detection circuit processes *I_i_* and obtains the feedback current *I_o_*, which is outputted to the torque device, and under the action of the torque device, the flexible pendulum returns to the equilibrium position, forming a closed-loop feedback system. The feedback current *I_o_* can reflect the acceleration *a*. It can be seen that the photosensor is at the source of accelerometer signal detection, and its detection accuracy is critical.

**Figure 2:**
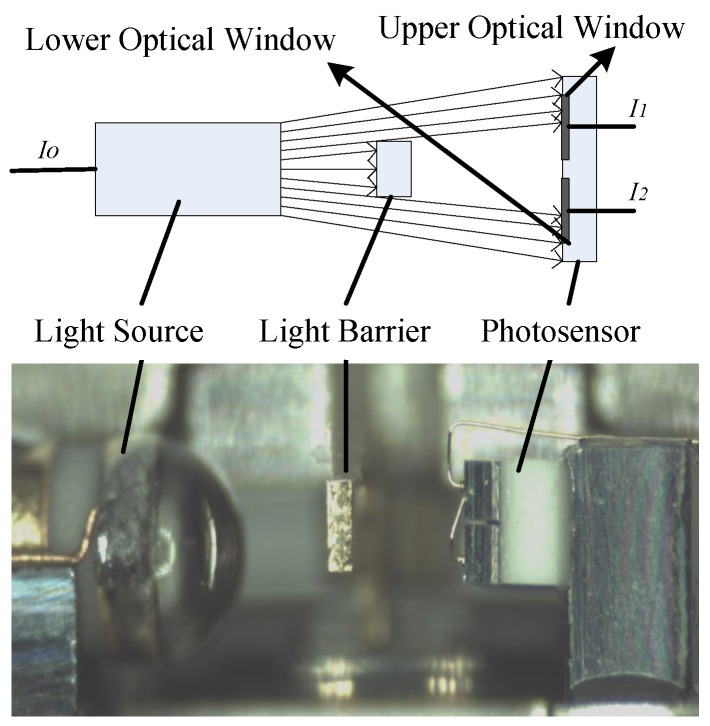
Composition of precision photosensor.

Figure 2 illustrates the components of the precision photosensor; it is composed of three main components: a light source, a light barrier, and a photosensor. The light source, powered by a current, emits infrared light at a constant frequency. Positioned centrally and integrated with the accelerometer’s flexible pendulum, the light barrier splits the infrared light from the source into two separate beams. These upper and lower beams irradiate the respective upper and lower optical windows of the photosensor. Each optical window then transforms the incident light intensity into induced currents, denoted as *I*_1_ and *I*_2_. The relationship between these induced currents (*I*_1_ and *I*_2_) and the light intensity *P* is linear and can be mathematically formulated as follows:

(1) $$\left\{\begin{array}{l} I_{1}=k_{1}P+B_{01}\\ I_{2}=k_{2}P+B_{02} \end{array}\right.$$

where $k_{1}$ and $k_{2}$ are the coefficient associated with the photosensor, and $B_{01}$ and $B_{02}$ are the zero bias of the induced current.

When the light barrier is aligned at the center of the light beam emitted by the source, the light intensities on both the upper and lower segments of the barrier are equal, resulting in equal induced currents (*I*_1_ and *I*_2_) post photosensitive conversion; hence, *I*_1_ = *I*_2_. If the light barrier tilts upwards, the light intensity in the upper portion decreases, leading to a condition where *I*_1_ is less than *I*_2_ after conversion. In the opposite scenario, where the light barrier tilts downwards, the light intensity in the upper portion increases, causing *I*_1_ to exceed *I*_2_ after photosensitive conversion. Based on this operating principle, any displacement of the baffle plate is translated into a difference Δ*I* between the currents *I*_1_ and *I*_2_. This difference Δ*I* serves as a precise measure of the accelerometer pendulum’s movement, allowing for the accurate tracking and assessment of its displacement.

(2) $$\Delta I=I_{2}-I_{1}=(k_{2}-k_{1})P+B_{02}-B_{01}$$

To implement the displacement detection scheme described, it is imperative that the outputs *I*_1_ and *I*_2_ from the photosensor remain stable, with the difference Δ*I* being zero when the light barrier is in a centered and in a stationary position. This necessitates the use of a highly accurate light source and photosensor. Nonetheless, the accuracy of the system can be compromised by external environmental factors, with temperature being particularly influential.

**Figure 3:**
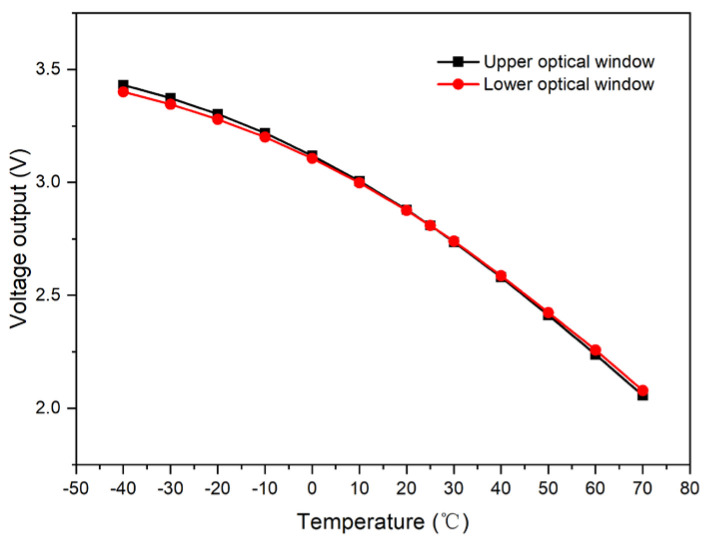
Upper and lower optical window output curves with respect to temperature.

Upon the removal of the photosensor’s light barrier, a signal acquisition circuit was designed for testing purposes. By driving the light source with a constant current of 3 mA across a range of temperatures, the currents at the upper and lower optical windows were amplified. These currents were then converted into voltage outputs, as illustrated in Figure 3.

When a constant drive current is applied, the light source’s intensity varies with temperature, leading to corresponding changes in the output curves of both the upper and lower optical windows, which typically show a decrease as temperature rises. Analysis indicates that temperature predominantly impacts the light source’s intensity, denoted as *P*. As depicted in Figure 3, the relationship between the light intensity *P* and temperature *T* is primarily quadratic and can be mathematically represented by the following equation:

(3) $$P=AT^{2}+BT+C$$

where *A*, *B* and *C* are all coefficients, which are determined by the characteristics of light intensity *P* changing with temperature.

Temperature fluctuations induce variations in the photosensor’s output, which in turn can compromise the precision of the light barrier’s displacement measurement and consequently diminish the accelerometer’s accuracy. Consequently, mitigating or negating the thermal impact on the photosensor’s output is essential for enhancing the accelerometer’s precision. This paper concentrates on developing a compensation strategy that adjusts the drive current to regulate light intensity, thereby curtailing the temperature’s influence on the photosensor’s output.

## 3. Open-Loop Compensation Scheme

**Figure 4:**
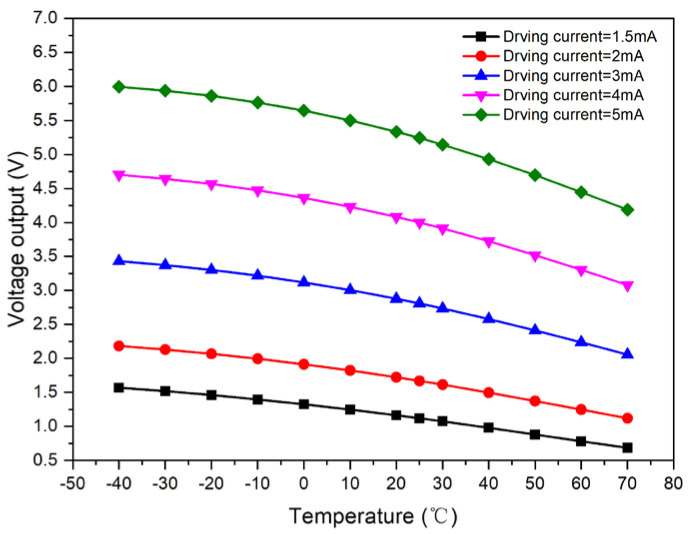
Test curve of Sample 1 with respect to temperature under different driving currents.

The driving current was incrementally set to 1.5, 2, 3, 4, and 5 mA and applied to Sample 1. Concurrently, the incubator temperature was meticulously regulated through a range of −40, −30, −20, −10, 0, 10, 20, 25, 30, 40, 50, 60, and 70 °C to ensure a consistent thermal setting. The resultant output curves of the upper optical window for Sample 1, plotted as a function of temperature across the various driving currents, are presented in Figure 4.

**Figure 5:**
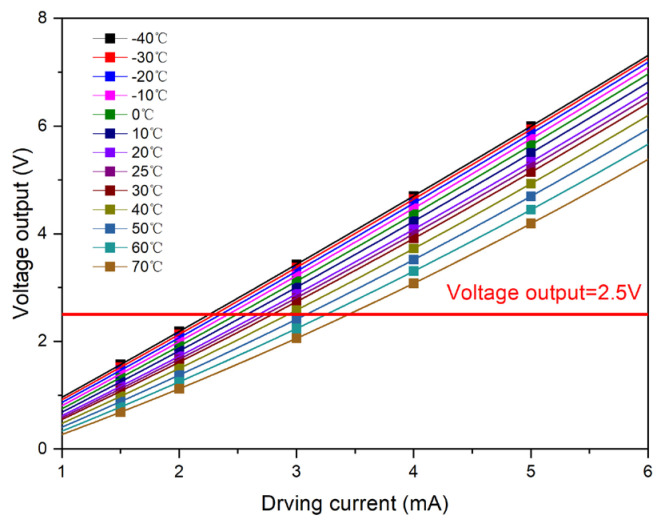
Test curve of Sample 1 with respect to the current at different temperatures.

Under varying driving currents, it was observed that an increase in temperature corresponded to a decrease in sensor output. Additionally, the trend of this change was not strictly linear. The output curves relative to the driving current at various temperatures were determined and are depicted in Figure 5, which illustrates the test curve for the output of the upper optical window of Sample 1.

**Figure 6:**
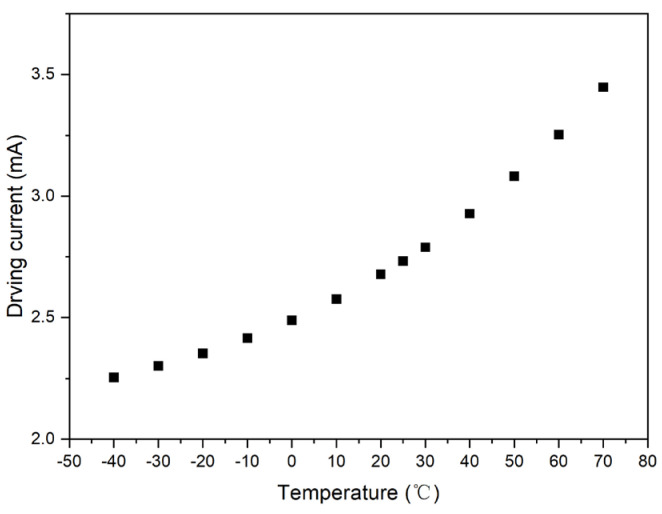
Ideal compensation curve of Sample 1.

The output value of the photosensor was set to a fixed value: *V*_0_ = 2.5 V, and the intersection point formed by the test curve and straight line, *V*_0_ = 2.5 V, was plotted. Accordingly, the driving current compensation curve with respect to temperature was obtained, as shown in Figure 6.

Assuming that the compensation at different temperatures is $\Delta I_{m}(T)$, the compensation relation can be expressed as

(4) $$\left\{\begin{array}{l} I_{1}^{\prime }=k_{1}P^{\prime }+B_{01}\\ P^{\prime }=P+\Delta I_{m}(T)\\ I_{1}^{\prime }=V_{0}/R \end{array}\right.$$

where $I_{1}^{\prime }$ is the output-induced current of the upper optical window after temperature compensation, *P*′ is the light intensity of the light source through the compensation, and *R* is the equivalent impedance of converting induced current into voltage.

Since the upper and lower light windows receive the same light intensity from the same light source, the induced current output from the lower light window is

(5) $$I_{2}^{\prime }=k_{2}P^{\prime }+B_{02}$$

The current difference after compensation is expressed as

(6) $$\Delta I^{\prime }=\left(k_{2}-k_{1}\right)P^{\prime }+B_{02}-B_{01}.$$

Comparing $\Delta I^{\prime }$ and ΔI, the current difference between pre-compensation and post-compensation mainly depends on the light intensity *P*′ and *P*. Assuming that the temperature compensation reaches the ideal state, the light intensity after compensation *P*′ stays constant with the temperature, while the light intensity *P* before compensation varies with the temperature, and therefore, the interval of the current difference with temperature after compensation is necessarily smaller than before compensation.

(7) $${\left|\Delta I^{\prime }\right|}_{\max}\leq {\left|\Delta I\right|}_{\max}$$

where ${\left|\Delta I^{\prime }\right|}_{\max}$ is the total temperature change of the current difference after compensation, and ${\left|\Delta I\right|}_{\max}$ is the total temperature change of the current difference before compensation.

Perfect temperature compensation cannot be achieved in practical engineering applications. From Equation (6), it can be seen that the final result of the output $\Delta I^{\prime }$ mainly depends on *P*′. From Equations (4) and (5), it can also be seen that the outputs of the upper and lower light window $I_{1}^{\prime }$ and $I_{2}^{\prime }$ are also dependent on *P*′. Therefore, by analyzing the effect of the compensation of the upper light window, $I_{1}^{\prime }$ can be used as a substitute for the analysis of the compensation of the current difference $\Delta I^{\prime }$.

If the variance fitted by the drive current compensation curve is considered, the variance *S* of the actual driving current compensation $\Delta I_{m}^{\prime }(T)$ after fitting by the single-window least-square method can be expressed as

(8) $$S=\sum_{i=1}^{n}{\left(\Delta I_{m}^{\prime }(T_{i})-\Delta I_{m}(T_{i})\right)}^{2}$$

where $T_{i}$ is the temperature point *i*, $\Delta I_{m}^{\prime }(T_{i})$ is the driving current compensation corresponding to the temperature point *i*, and $\Delta I_{m}(T_{i})$ is the ideal driving current compensation corresponding to the temperature point *i* in Figure 6. Therefore, the actual driving current compensation $\Delta I_{m}^{\prime }(T)$ can be expressed as

(9) $$\Delta I_{m}^{\prime }(T)=\Delta I_{m}(T)+e(T)$$

where e(T) is the difference between the actual driving current compensation and the ideal driving current compensation, and its size is determined by the variance *S* fitted by the driving current compensation $\Delta I_{m}(T)$ least-square method. The greater the variance *S*, the greater the variance e(T), and the smaller the variance *S*, the smaller the variance e(T). Therefore, the quality of the compensated output current difference can be measured by fitting the variance *S* with the temperature compensation of a single window. A larger *S* indicates that the error between the actual driving current compensation $\Delta I_{m}^{\prime }(T)$ and the ideal driving current compensation $\Delta I_{m}(T)$ is larger, and the fluctuation is larger; a smaller *S* indicates that the error between the actual driving current compensation $\Delta I_{m}^{\prime }(T)$ and the ideal driving current compensation $\Delta I_{m}(T)$ is smaller, and the compensated light intensity *P’* also approaches a straight line, and the fluctuation of $\Delta I^{\prime }$ is smaller.

In addition, in practical engineering applications, the characteristics of the same types of photosensor are relatively similar, but individual differences are present. To compensate more photosensors to the qualified range, it is necessary to have more data of samples to solve the batchwise problem. Therefore, eight photosensors were selected as samples for this study. The samples were thoroughly tested and compensated under different driving current and temperature conditions.

### 3.1. First-Order Compensation Scheme

**Figure 7:**
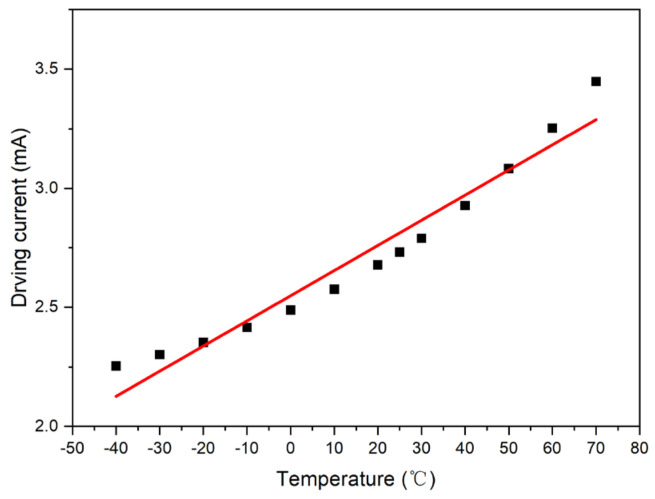
First-order compensation curve of Sample 1.

The first-order compensation model, *I_o_* = *A*_1_ × *T* + *B*_1_, can be obtained by performing linear least-square fitting of the Sample 1 model, as shown in Figure 7. The fitting parameters were calculated as *A*_1_ = 0.010132 and *B*_1_ = 2.563878.

As previously stated, in practical engineering applications, it is impractical and cost-prohibitive to develop a unique model for each photosensor due to their inherent individual variations. Therefore, a unified model was created to ensure that satisfactory compensation effects can be consistently achieved across different photosensors.

**Figure 8:**
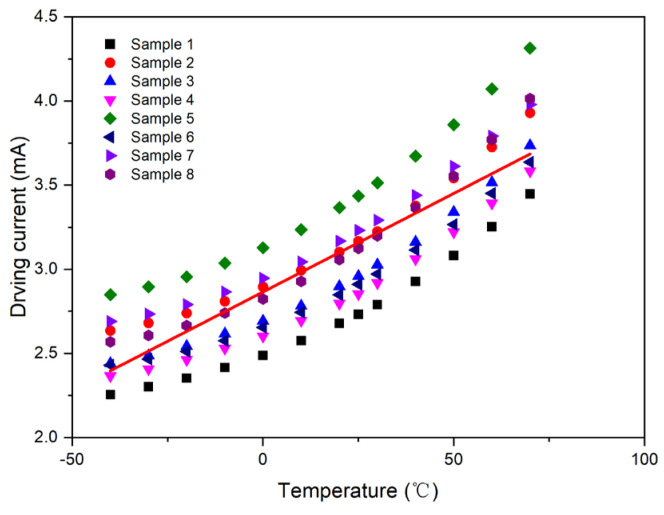
First-order compensation curve for Samples 1–8.

Consequently, a unified first-order compensation model was derived through the linear least-squares fitting of the collective sample data, as depicted in Figure 8. The resulting parameters from the fitting process were *A*_1_ = 0.011112 and *B*_1_ = 2.864365.

Table 1 presents the parameters for both the individual first-order compensation models and the unified first-order compensation model for each sample, based on the fitting outcomes.

**Figure 9:**
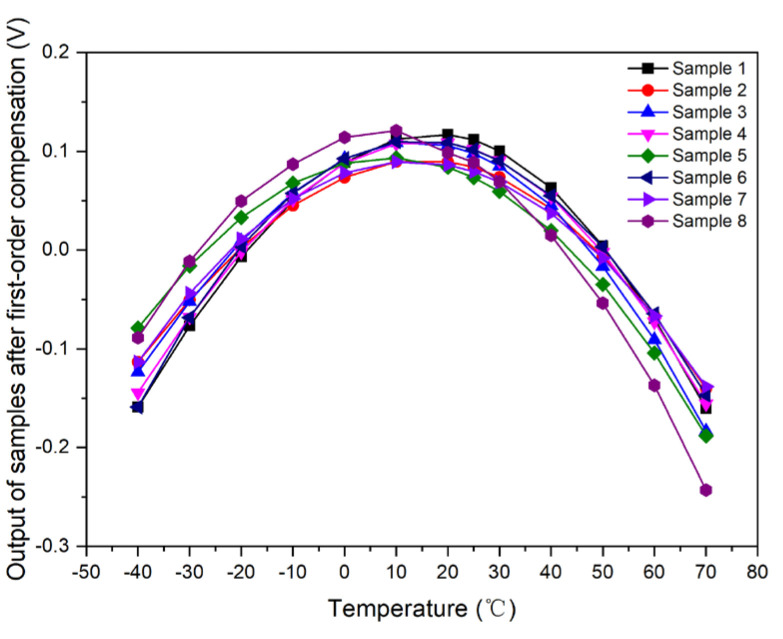
Output curve of samples with respect to temperature after first-order compensation.

Samples 1–8 used the unified first-order compensation model for compensation; that is, *I_o_* = 0.011112 × *T* + 2.864365. The output curve with respect to temperature was simulated and calculated after data normalization, as shown in Figure 9.

**Figure 10:**
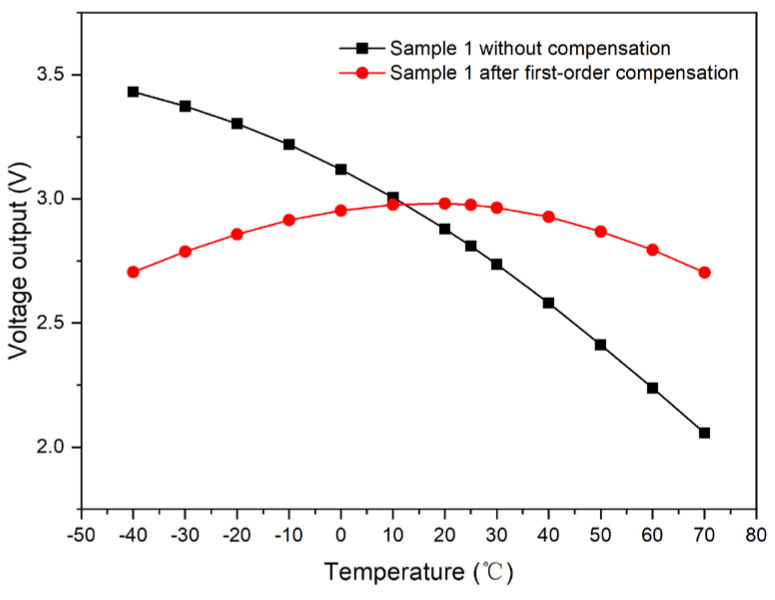
Comparison of output curves of Sample 1 with respect to the temperature before and after first-order compensation.

Taking Sample 1 as an illustrative case, we compared the light-intensity output voltage in the uncompensated state to that after the application of first-order compensation. Figure 10 indicates that the voltage range without compensation is from 2.05737 to 3.43162 V. In contrast, the range is narrowed to 2.70401 to 2.98143 V after first-order compensation, resulting in a substantial reduction of 79.8%. This demonstrates that the compensation scheme has a significant impact on the output voltage stability and accuracy.

**Figure 11:**
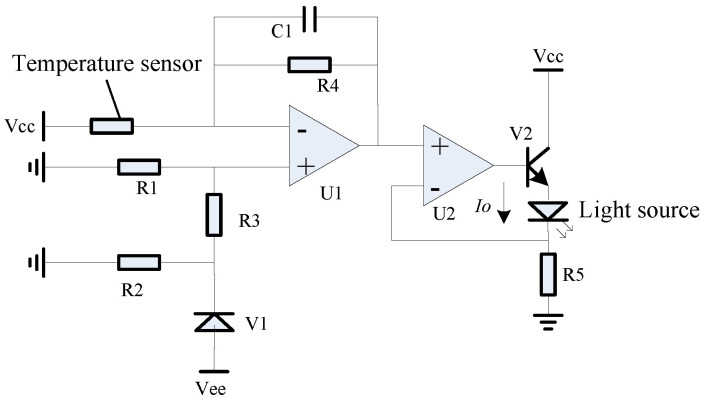
Schematic circuit diagram of the first-order compensation scheme.

The hardware circuit, as depicted in Figure 11, was crafted in alignment with the first-order compensation scheme. A temperature sensor was employed to gather temperature data as the input for the system. A stable voltage reference was established using the voltage reference diode V1, along with resistors R1 and R3. Operational amplifiers U1 and R4 were used to achieve amplification and linear calculations. Finally, the combined current source from operational amplifier U2, power tube V2, and resistor R5 generated the feedback drive current *I_o_*, for driving the light source to achieve the compensation function.

### 3.2. Second-Order Compensation Scheme

**Figure 12:**
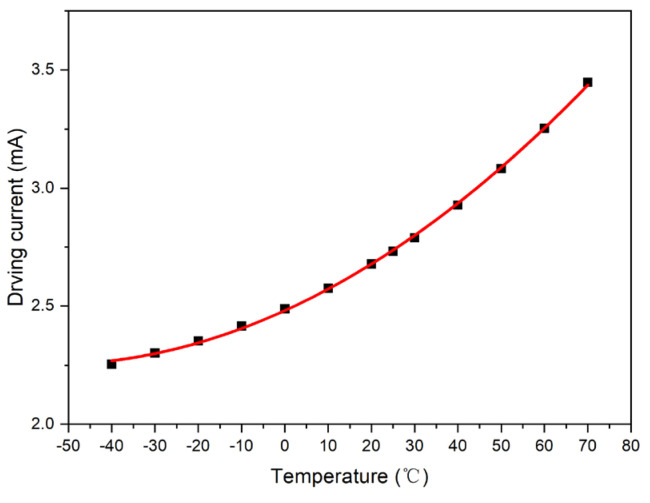
Second-order compensation curve of Sample 1.

To further improve the compensation accuracy, the ideal model of Sample 1 (Figure 6) was fitted using a second-order least-square method. Moreover, a second-order compensation scheme was designed. Furthermore, model *I_o_ = A*_2_ *× T*^2^ *+ B*_2_ *× T + C*_2_ was established, from which a new model was obtained, as shown in Figure 12. The fitting values are as follows: *A*_2_ = 7.34 × 10^−5^, *B*_2_ = 0.00847, and *C*_2_ = 2.481291.

**Figure 13:**
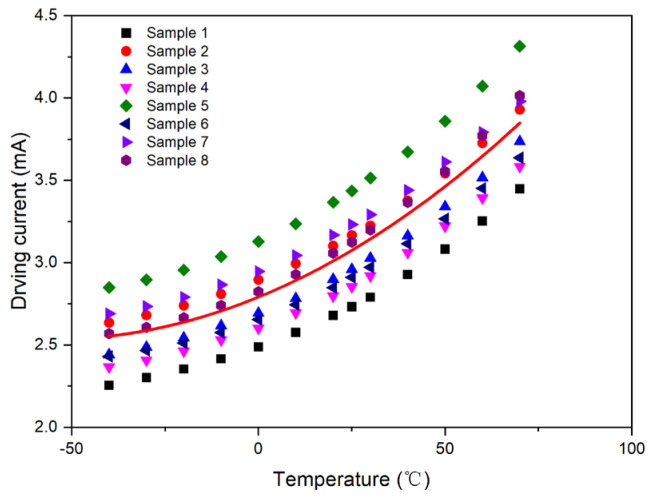
Second-order compensation curve for Samples 1–8.

Similarly, a unified second-order compensation model was obtained by fitting all compensation models of the eight samples with second-order polynomials, as shown in Figure 13. The fitting results are as follows: *A*_2_ = 8.12 × 10^−5^, *B*_2_ = 0.009273, and *C*_2_ = 2.772991.

Table 2 lists the parameters of the individual second-order compensation model and unified second-order compensation model for each sample according to the fitting results.

**Figure 14:**
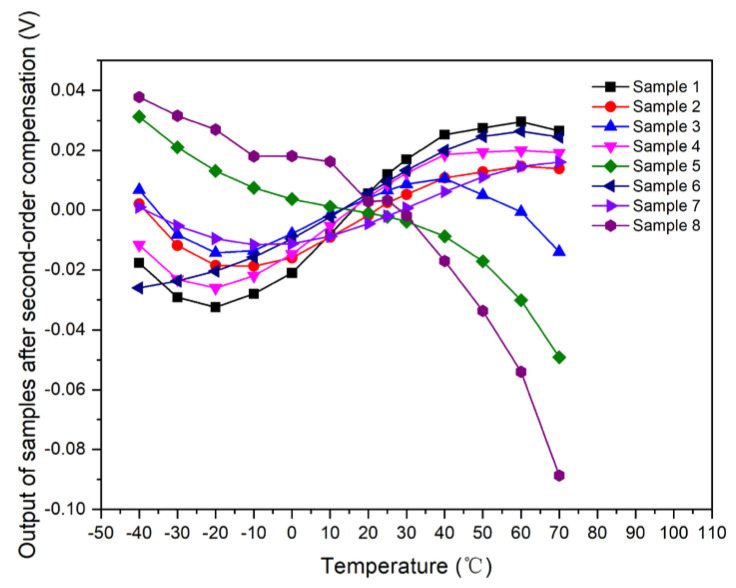
Output curve of samples with respect to temperature after second-order compensation.

Samples 1–8 used these unified second-order compensation model for compensation; that is, *I_o_* = 8.12 × 10^−5^ × *T*^2^ + 0.009273 × *T* + 2.772991. The output curve with respect to temperature was simulated and calculated after data normalization, as shown in Figure 14.

**Figure 15:**
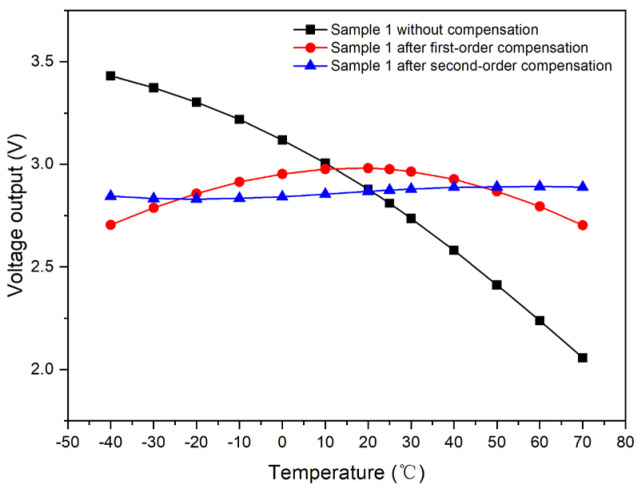
Comparison of output curves of Sample 1 with respect to temperature before and after second-order compensation.

Taking Sample 1 as a reference, we can evaluate the light-intensity output voltage in three conditions: without any compensation, after first-order compensation, and after second-order compensation. Figure 15 illustrates these comparisons.

The voltage range after second-order compensation is from 2.83044 to 2.89242 V. This range is 77.7% narrower than the range post first-order compensation and 95.5% narrower than the uncompensated range. Hence, the second-order compensation yields a more pronounced effect, significantly reducing the voltage fluctuation.

**Figure 16:**
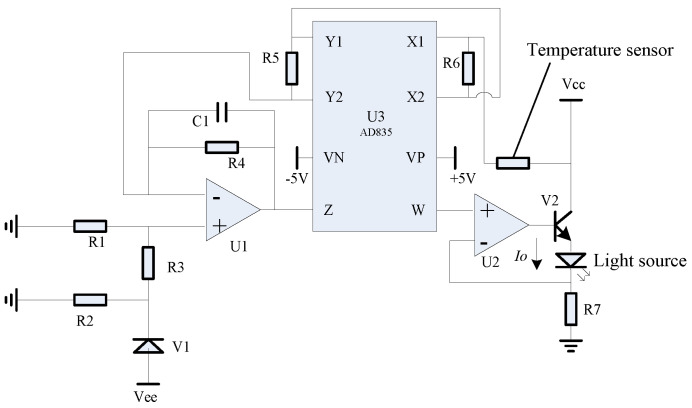
Schematic circuit diagram of second-order compensation scheme.

The hardware circuit, as detailed in Figure 16, was engineered in accordance with the second-order compensation scheme. It utilizes a multiplier AD835-based linear circuit at its core. The temperature sensor signals are processed and then directed to the multiplier, which outputs the product to terminals X1, X2, Y1, and Y2. Subsequently, the second-order, first-order, and constant components of the compensation model are integrated after their respective signals are connected to terminal Z. The culmination of this circuit is the generation of a feedback drive current *I_o_*, through the synergistic action of operational amplifier U2, power tube V2, and resistor R7.

### 3.3. Third-Order Compensation Scheme

**Figure 17:**
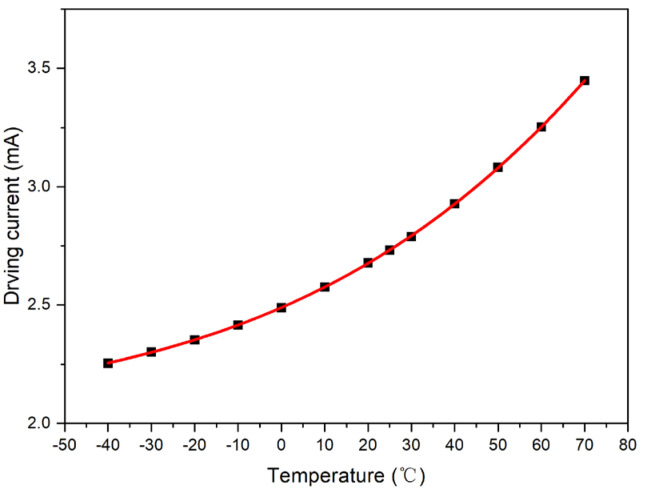
Third-order compensation curve of Sample 1.

The ideal model of Sample 1 (shown in Figure 6) was fitted with a third-order polynomial. Moreover, a third-order compensation scheme was designed. Furthermore, model *I_o_ = A*_3_ *× T*^3^ *+ B*_3_ *× T*^2^ *+ C*_3_ *× T + D*_3_ was established, from which a new model was obtained, as shown in Figure 17. The fitting results are as follows: *A*_3_ = 2.81 × 10^−7^, *B*_3_ = 6.22 × 10^−5^, *C*_3_ = 0.007977, and *D*_3_ = 2.490108.

**Figure 18:**
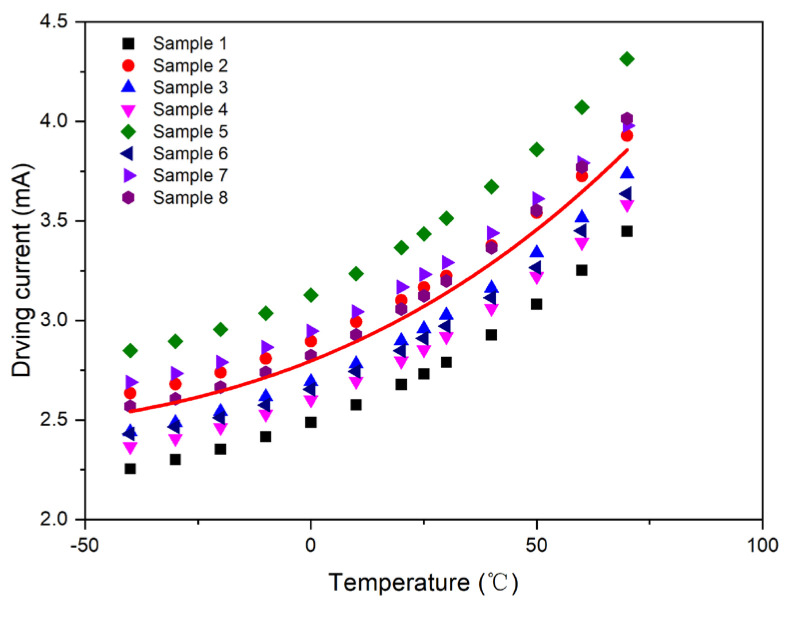
Third-order compensation curve for Samples 1–8.

Moreover, a unified third-order compensation model was obtained by fitting all compensation models of the eight samples with third-order polynomials, as shown in Figure 18. The fitting results are as follows: *A*_3_ = 2.01 × 10^−7^, *B*_3_ = 7.32 × 10^−5^, *C*_3_ = 0.008919, and *D*_3_ = 2.779314.

Table 3 details the parameters for both the individual and unified third-order compensation models for each sample, as derived from the fitting outcomes.

**Figure 19:**
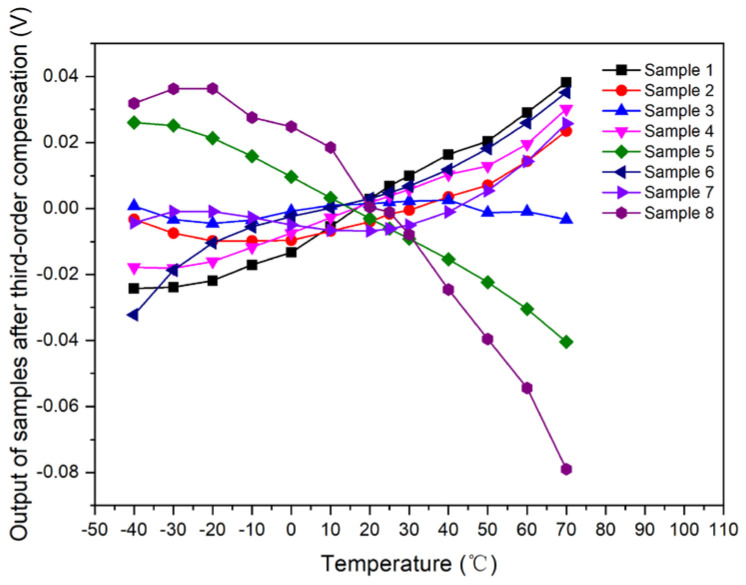
Output curve of samples with respect to temperature after third-order compensation.

Samples 1–8 used these unified third-order compensation model for compensation; that is, *I_o_* = 2.01 × 10^−7^ × *T*^3^ + 7.32 × 10^−5^ × *T*^2^ + 0.008919 × *T* + 2.779314. The output curve with respect to temperature was simulated and calculated after data normalization, as shown in Figure 19.

**Figure 20:**
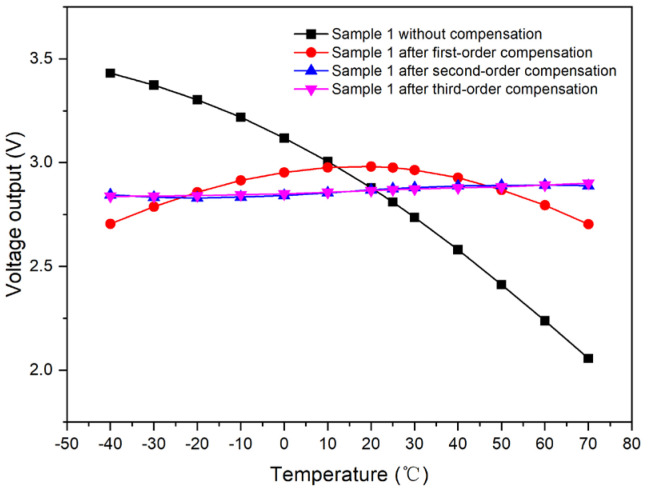
Comparison of output curves of Sample 1 with respect to temperature before and after third-order compensation.

Taking Sample 1 as a reference, we compared the light-intensity output voltage without any compensation to that after applying first-order, second-order, and third-order compensations. As illustrated in Figure 20, the voltage range after third-order compensation is between 2.83864 and 2.9011 V. This range is 0.77% higher than the second-order compensation but 77.5% and 95.5% lower than the first-order compensation and the uncompensated state, respectively. Hence, the effect of third-order compensation is akin to that of the second-order scheme.

Repeated multiplication is essential for the implementation of the third-order compensation scheme, which makes its circuit design more complex than those of the first- and second-order compensation models.

In conclusion, the first-order, second-order, and third-order compensation schemes have been demonstrated to effectively compensate for photosensors. The compensation errors were determined by comparing the output of each sample, compensated according to the different schemes, with the output from an ideal compensation scheme, as detailed in Table 4.

The compensation errors, post-adjustment, for the first-order, second-order, and third-order schemes were estimated to be approximately 0.093169, 0.018779, and 0.01686, respectively. While the second- and third-order schemes demonstrated superior compensation efficacy, the enhancement offered by the third-order scheme over the second was not markedly significant. In terms of circuit complexity and implementation, the first-order scheme’s circuit was the most straightforward, making it the easiest to execute. The second-order scheme presented a moderate level of complexity in its circuitry, whereas the third-order scheme was the most intricate. Considering practical applications, the first-order scheme is well-suited for environments that demand a moderate level of compensation accuracy coupled with a straightforward circuit scale. The second-order scheme is ideal for scenarios that prioritize high compensation accuracy and can accommodate a more complex circuit scale. However, the third-order scheme, with its third-order term coefficient ranging from 10^−7^ to 10^−8^, was deemed less advantageous than the second-order approach and, as a result, was not deemed suitable for application in this context.

The first- and second-order compensation schemes operate by taking the temperature signal as input and adjusting the drive current as output, respectively. They rely on a set model for compensation, characterizing them as open-loop methods. However, for photosensors that exhibit poor consistency, the use of a static model might not suffice to meet application requirements, as it may not adequately address the variability in their behavior. Furthermore, these compensation schemes, being primarily focused on temperature effects, may fall short when it comes to mitigating the impacts of other factors. They are not designed to provide comprehensive compensation for influences beyond temperature, such as magnetic fields, mechanical stress, or aging effects, which can also significantly affect the performance of photosensors. As a result, while the first- and second-order schemes offer a structured approach to temperature compensation, they may require augmentation or integration with other techniques to achieve a more holistic compensation for photosensors in diverse and unpredictable environments.

## 4. Closed-Loop Compensation Scheme

**Figure 21:**
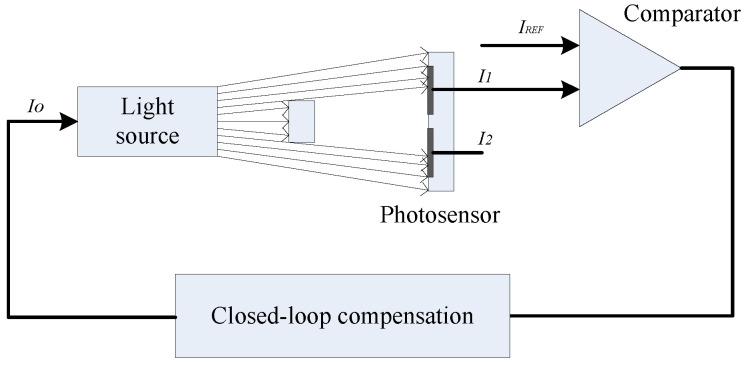
Operating principle of closed-loop compensation scheme.

A closed-loop compensation scheme, as delineated in Figure 21 and grounded in the principles of automatic control, has been crafted. This scheme employs a target reference current, $I_{REF}$, which is utilized for continuous, real-time comparison against the output current, *I*_1_, derived from the upper optical window of the photosensor. If the condition *I*_1_ > $I_{REF}$ is met, the driving current for the light source, *I_o_*, is appropriately reduced. Conversely, if *I*_1_ < $I_{REF}$, *I_o_* is incremented. Should *I*_1_ be equal to $I_{REF}$, the driving current remains stable. This sophisticated arrangement allows for the dynamic monitoring and adjustment of the light intensity detected by both the upper and lower optical windows of the photosensor. In response to temperature fluctuations, the values of *I*_1_ and *I*_2_ are bound to shift; the closed-loop system promptly adjusts the driving current Io to counteract these changes, ensuring that the light source’s intensity is modified to restore *I*_1_ to the desired $I_{REF}$ level. Furthermore, the system is adept at addressing the degradation in photosensor performance that can occur due to prolonged operation or other environmental factors. The closed-loop compensation mechanism ensures that *I*_1_ and *I*_2_ are rectified to their requisite values, thereby maintaining the photosensor’s output integrity and reliability over time. This adaptive capability is a testament to the robustness and accuracy afforded by the closed-loop compensation scheme in navigating the intricate dynamics of photosensor operation.

**Figure 22:**
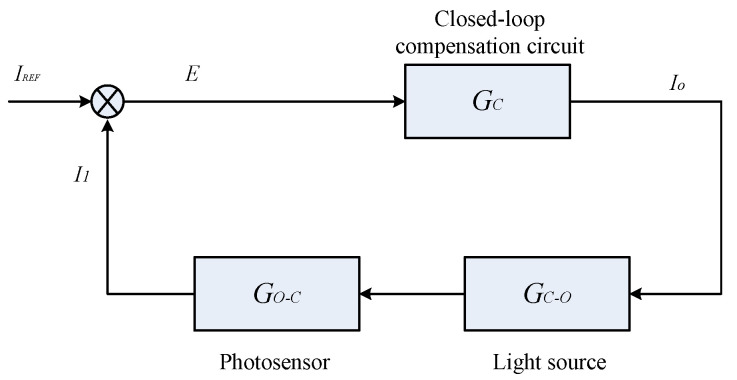
Control block diagram of closed-loop compensation.

The control block diagram, as outlined for the aforementioned closed-loop compensation scheme and presented in Figure 22, illustrates the system’s operational flow. $I_{REF}$ represents the preset reference current, while *I*_1_ signifies the output current from the photosensor’s upper optical window. The variable *E* represents the differential value between these two currents. When these currents are fed into the compensation circuit, they are governed by the transfer function $G_{c}$. The resultant drive current *I_o_*, which powers and illuminates the light source, is determined by another transfer function, denoted as $G_{c-o}$. The photosensor’s role in transforming the incoming light into an electrical current output is described by the conversion relationship $G_{o-c}$. This closed-loop system is designed to dynamically adjust and stabilize the photosensor’s performance, ensuring optimal output irrespective of environmental variations.

The error of the closed-loop system can be calculated using the following equation:

(10) $$E=\frac{I_{REF}}{\left(1+G_{c}\cdot G_{c-o}\cdot G_{o-c}\right)}.$$

This control system ensures that *I*_1_ equals $I_{REF}$ at all times; that is, *E* = 0. Thus, designing an integrator in the closed-loop circuit to form a system for achieving no static error is necessary considering that the function of the system is a proportional link.

**Figure 23:**
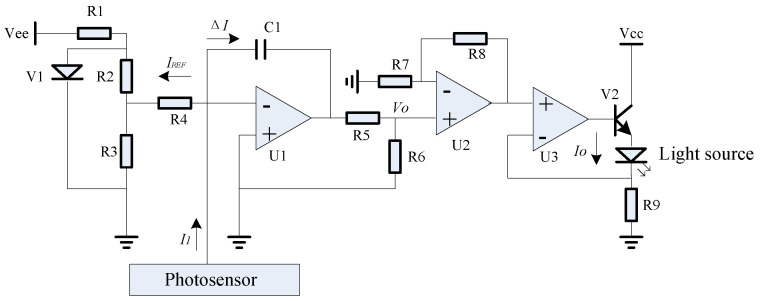
Schematic circuit diagram of closed-loop compensation scheme.

The analog circuit, as depicted in Figure 23, was crafted to implement the aforementioned closed-loop compensation scheme. It utilized the voltage reference V1 alongside resistors R2, R3, and R4 to produce the reference current, denoted as $I_{REF}$. The current *I*_1_, emanating from the photosensor’s upper optical window, was offset by $I_{REF}$, yielding a difference signal. This difference was then integrated by the operational amplifier U1 and capacitor C1, resulting in the formation of the feedback voltage *V_o_*. Subsequently, this voltage was amplified by the components U2, R7, and R8. Ultimately, the feedback drive current *I_o_* was generated by the confluence of operational amplifier U3, power tube V2, and resistor R9, which in turn drove the light source, thereby accomplishing closed-loop control.

Compared to the first- and second-order compensation schemes, the closed-loop compensation scheme is capable of adjusting the output drive current through closed-loop control, ensuring that the output signal from the upper optical window of the photosensor matches the set value in real time. In order to ensure that the effect of closed-loop compensation is better than open-loop temperature compensation, the closed-loop compensation is designed to control the output error of the photosensor within 1 mV. Therefore, in this paper, the response time or compensation period of the closed-loop circuit is about 0.1 s, and the output error of the photosensor can be theoretically controlled at 0.227 mV. In the engineering, this is realized by designing and adjusting the integrating capacitance C1 and the resistors R5, R6, R7, R8, R9 in the closed-loop compensation circuit.

Additionally, unlike the open-loop compensation scheme, the closed-loop scheme does not require the conversion of temperature information, which simplifies the operating principle and enhances compensation accuracy. Each photosensor has its own independent compensation model to achieve precise control. Moreover, the closed-loop compensation scheme can account for the effects of time and other factors on the photosensor, in addition to temperature influences.

## 5. Experimental Verification

To verify the feasibility of various compensation models and compare the compensation effects of various models, grouped experiments were designed as follows:

1. Combine photosensors 1 and 2 with the first-order compensation circuit into experimental Samples A1 and A2. Set the incubator temperature within the range of −40 to 70 °C and test the output voltage of the photosensor under different temperatures.
2. Combine photosensors 1 and 2 with the second-order compensation circuit into experimental Samples B1 and B2. Set the incubator temperature within the range of −40 to 70 °C and test the output voltage of the photosensor under different temperatures.
3. Combine photosensors 1 and 2 with the closed-loop compensation circuit into experimental Samples C1 and C2. Set the incubator temperature within the range of −40 to 70 °C and test the output voltage of the photosensor under different temperatures.

**Figure 24:**
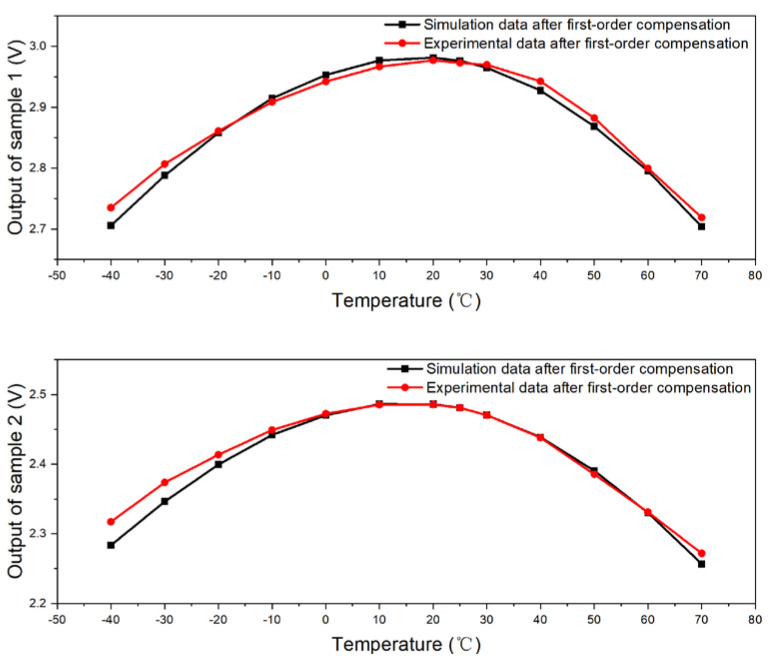
Comparison between experimental and simulation data (Samples 1 and 2 after first-order compensation).

Figure 24 offers a visual synthesis that contrasts the empirical data from Samples A1 and A2 in the group (1) experiment against the simulation data for sensors 1 and 2. These simulations are predicated on the first-order compensation model, which is designed to adjust for temperature-induced variations. The figure illustrates a remarkable alignment between the experimental data and the simulation, indicating that the first-order model closely mirrors the samples’ behavior across a range of temperatures. The discrepancies between the experimental and simulated data are quantified, with the maximum errors for A1 and A2 recorded at approximately 0.0296 V and 0.0336 V, respectively. When these errors are expressed as a percentage of the overall output, they correspond to relative errors of about 1% for A1 and 1.3% for A2. These figures, while modest, provide a clear indication of the accuracy of the first-order compensation model in predicting the thermal response of the photosensors.

**Figure 25:**
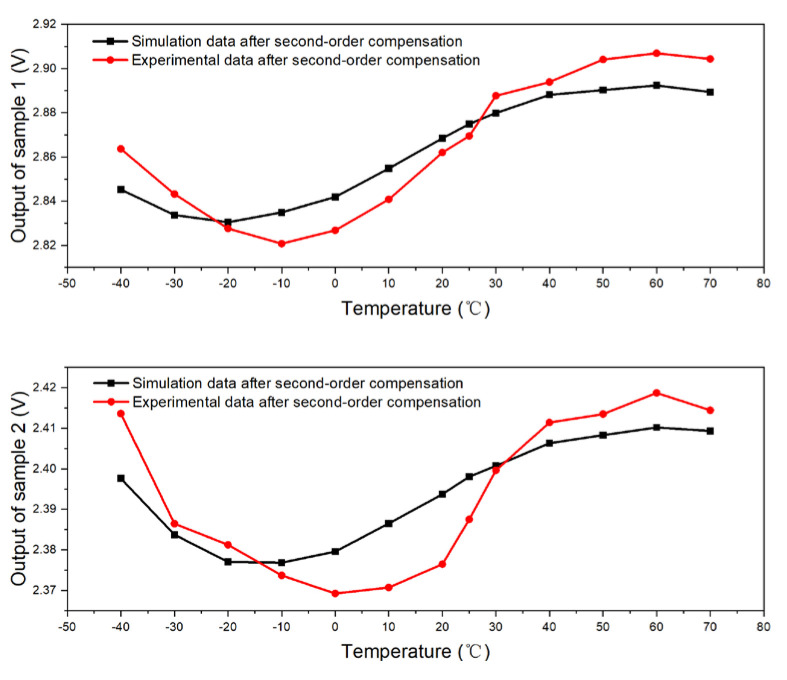
Comparison between experimental and simulation data (Samples 1 and 2 after second-order compensation).

Figure 25 presents a comparative analysis of the empirical data garnered from Samples B1 and B2 in the group (2) experiment, juxtaposed against the simulated data predicated on the second-order compensation model for sensors 1 and 2. The congruence between the experimental and simulated data across varying temperatures is strikingly evident, indicating a high degree of fidelity in the predictive model. The maximum deviations observed between the experimental outputs of B1 and B2, and their corresponding simulated counterparts, are calculated to be approximately 0.0184 V and 0.0172 V, respectively. These discrepancies, when measured relative to the overall performance, translate to relative errors of about 0.64% for B1 and 0.72% for B2. Such marginal errors not only substantiate the accuracy of the second-order compensation model but also reflect the robustness of the experimental setup in mirroring the simulated conditions.

**Figure 26:**
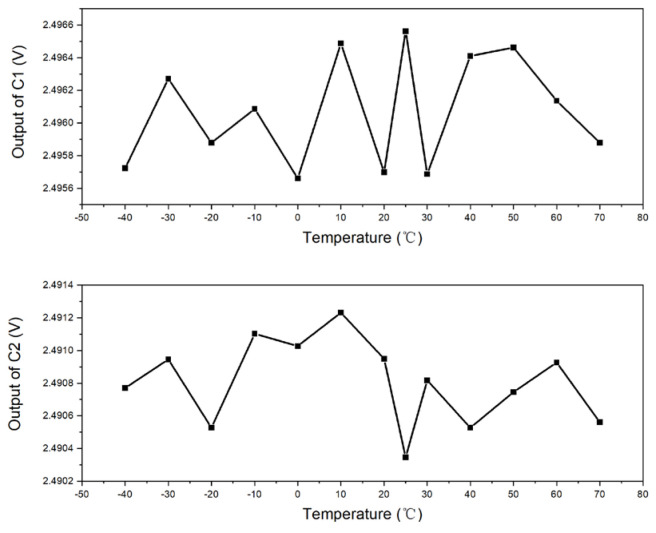
Experimental data of Samples 1 and 2 after closed-loop compensation.

The graphical representation of the performance variance under varying thermal conditions was meticulously extracted from the dataset of Samples C1 and C2 within the third experimental group, as illustrated in Figure 26. The output readings for C1 and C2 are delineated within a narrow range, with C1 fluctuating between 2.49656 and 2.49566, and C2 from 2.49123 to 2.49034. Upon a meticulous evaluation of the average values, the maximum and proportional discrepancies for C1 were ascertained to be roughly 0.00049 and 0.0196%, respectively. Similarly, for C2, these values were determined to be approximately 0.00046 and 0.0185%, respectively. These subtle differences underscore the high degree of accuracy and the marginal error margin inherent in the samples’ responses to temperature variations.

**Figure 27:**
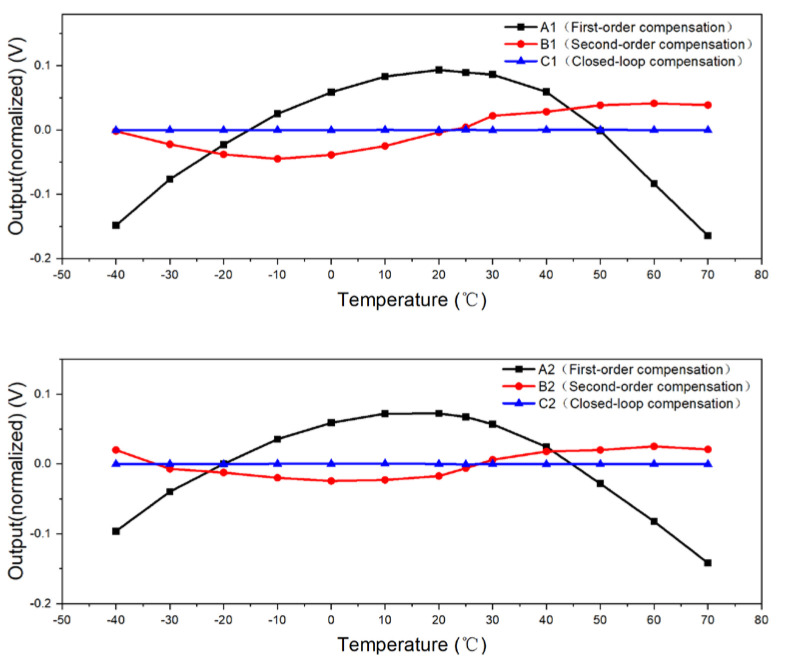
Comparison between experimental data after different compensations.

The comparative visualization of performance across various compensation circuits for photosensor 1 was achieved through the normalization of data from circuits A1, B1, and C1, yielding a clear contrast in outcomes. Employing an analogous method, the performance comparison for photosensor 2 was derived from the normalized data of circuits A2, B2, and C2. As depicted in Figure 27, the output voltages at each temperature checkpoint for A1, B1, and C1 were recorded at 0.258 V, 0.0862 V, and 0.0009 V, respectively. In contrast, the corresponding values for A2, B2, and C2 were observed to be 0.2141 V, 0.0495 V, and 0.0009 V, respectively.

The simulation and experimental results can be summarized as follows:

(1) The performance of photosensors, when integrated with various compensation circuits, was found to align closely with our simulation predictions. For the first-order temperature compensation, the discrepancy between experimental and simulated results was a minimal 1%, highlighting the model’s reliability. The second-order temperature compensation demonstrated even greater congruence, with a relative error reduced to approximately 0.7%. Most notably, the closed-loop compensation showcased exceptional stability, with a mere fluctuation error of about 0.02%, underscoring its superior accuracy.
(2) The efficacy of compensation circuits varied significantly. The second-order compensation outperformed the first-order compensation, yielding an output range that was roughly 70% broader across various temperature points. However, the closed-loop compensation distinguished itself as the most effective, with an output range that expanded by an impressive 98% relative to that achieved by second-order compensation. This marked enhancement illustrates the closed-loop system’s unparalleled capacity to optimize photosensor performance amidst varying temperatures.

## 6. Conclusions

In our research, we delved into two pivotal compensation methodologies—open-loop and closed-loop—to mitigate the impact of temperature fluctuations on photosensor outputs. A meticulous examination was conducted on the open-loop method’s first- and second-order temperature compensation schemes. The first-order scheme is underpinned by a straightforward linear model, readily implementable through a basic linear circuit. This approach boasts a simple concept, compact circuitry, and manageable engineering execution. It excels in compensating for linear sensors but falls short with nonlinear ones, incurring an error of approximately 0.093169 V for the photosensor examined in this study. The second-order scheme, in contrast, demonstrates superior compensation for nonlinear sensors, as evidenced by our findings, with a significantly reduced error of about 0.018779 V. However, this refinement comes at the cost of increased complexity in circuit design and heightened precision demands on the constituent components, making it a more challenging endeavor. Introducing the innovative closed-loop compensation scheme, our study presents a paradigm shift. This scheme surpasses its predecessors by providing real-time monitoring of the photosensor output, coupled with an automatic control system that modulates the driving current and light source intensity. This closed-loop system stands out for its independence from the temperature-induced characteristic variations of photosensors, eschewing the need for temperature sensor signals and instead relying on self-regulation for compensation. Our results indicate that the closed-loop scheme remarkably enhances compensation efficacy by nearly 98% over the second-order scheme. It employs adaptable and flexible compensation models, forgoing rigid mathematical formulations in favor of tailored approaches for each photosensor, ensuring precise control. Furthermore, this scheme’s adaptability extends to compensating for a myriad of influences, including temperature, magnetic fields, temporal drift, and humidity, offering a holistic compensation strategy. The proposed closed-loop compensation scheme is not only poised for deployment in similar high-precision photosensor applications but also serves as a robust framework for future studies aimed at expanding the horizons of photosensor compensation capabilities.

## Figures and Tables

**Table 1:** Parameters of first order compensation model.

Samples	*A*_1_ (mA/°C)	*B*_1_ (mA)
1	0.010132	2.563878
2	0.011109	2.973447
3	0.010993	2.774164
4	0.010438	2.681457
5	0.012474	3.224654
6	0.010337	2.737464
7	0.011165	3.032288
8	0.012248	2.927566
Unified first-order compensation model	0.011112	2.864365

**Table 2:** Parameters of second-order compensation model.

Samples	*A*_2_ (mA/°C^2^)	*B*_2_ (mA/°C)	*C*_2_ (mA)
1	7.34 × 10^−5^	0.00847	2.481291
2	7.65 × 10^−5^	0.009377	2.887383
3	7.96 × 10^−5^	0.009191	2.684638
4	7.48 × 10^−5^	0.008744	2.597276
5	9.20 × 10^−5^	0.01039	3.12113
6	7.88 × 10^−5^	0.008552	2.648796
7	7.63 × 10^−5^	0.009437	2.946443
8	9.83 × 10^−5^	0.010022	2.81697
Unified fitting	8.12 × 10^−5^	0.009273	2.772991

**Table 3:** Parameters of third-order compensation model.

Samples	*A*_3_ (mA/°C^3^)	*B*_3_ (mA/°C^2^)	*C*_3_ (mA/°C)	*D*_3_ (mA)
1	2.81 × 10^−7^	6.22 × 10^−5^	0.007977	2.490108
2	2.33 × 10^−7^	6.72 × 10^−5^	0.008968	2.894698
3	2.88 × 10^−7^	6.81 × 10^−5^	0.008685	2.693677
4	2.05 × 10^−7^	6.66 × 10^−5^	0.008384	2.603716
5	2.12 × 10^−7^	8.35 × 10^−5^	0.010018	3.127788
6	1.02 × 10^−7^	7.47 × 10^−5^	0.008374	2.651991
7	5.35 × 10^−8^	7.42 × 10^−5^	0.009343	2.948124
8	2.37 × 10^−7^	8.88 × 10^−5^	0.009606	2.824412
Unified fitting	2.01 × 10^−7^	7.32 × 10^−5^	0.008919	2.779314

**Table 4:** Comparison between compensation errors of various models.

Samples	First-Order Compensation Error	Second-Order Compensation Error	Third-Order Compensation Error
1	0.10108	0.02402	0.020927
2	0.07961	0.012515	0.010154
3	0.09682	0.009154	0.002442
4	0.095022	0.017801	0.015034
5	0.087414	0.020979	0.02141
6	0.095871	0.019445	0.018
7	0.078463	0.009626	0.009575
8	0.111069	0.036693	0.037336
Mean value of compensation errors	0.093169	0.018779	0.01686

## Data Availability

The original contributions presented in the study are included in the article, further inquiries can be directed to the corresponding author.
